# Preterm Gut Microbiome Depending on Feeding Type: Significance of Donor Human Milk

**DOI:** 10.3389/fmicb.2018.01376

**Published:** 2018-06-27

**Authors:** Anna Parra-Llorca, María Gormaz, Cristina Alcántara, María Cernada, Antonio Nuñez-Ramiro, Máximo Vento, Maria C. Collado

**Affiliations:** ^1^Neonatal Research Group, Health Research Institute La Fe, University and Polytechnic Hospital La Fe, Valencia, Spain; ^2^Division of Neonatology, University and Polytechnic Hospital La Fe, Valencia, Spain; ^3^Department of Biotechnology, Institute of Agrochemistry and Food Technology, Spanish National Research Council, Valencia, Spain

**Keywords:** preterm infant, their mother’s own milk, donor human milk, formula milk, intestinal colonization, microbiota

## Abstract

Preterm microbial colonization is affected by gestational age, antibiotic treatment, type of birth, but also by type of feeding. Breast milk has been acknowledged as the gold standard for human nutrition. In preterm infants breast milk has been associated with improved growth and cognitive development and a reduced risk of necrotizing enterocolitis and late onset sepsis. In the absence of their mother’s own milk (MOM), pasteurized donor human milk (DHM) could be the best available alternative due to its similarity to the former. However, little is known about the effect of DHM upon preterm microbiota and potential biological implications. Our objective was to determine the impact of DHM upon preterm gut microbiota admitted in a referral neonatal intensive care unit (NICU). A prospective observational cohort study in NICU of 69 neonates <32 weeks of gestation and with a birth weight ≤1,500 g was conducted. Neonates were classified in three groups according to feeding practices consisting in their MOM, DHM, or formula. Fecal samples were collected when full enteral feeding (defined as ≥150 cc/kg/day) was achieved. Gut microbiota composition was analyzed by 16S rRNA gene sequencing. Despite the higher variability, no differences in microbial diversity and richness were found, although feeding type significantly influenced the preterm microbiota composition and predictive functional profiles. Preterm infants fed MOM showed a significant greater presence of Bifidobacteriaceae and lower of Staphylococcaceae, Clostridiaceae, and Pasteurellaceae compared to preterm fed DHM. Formula fed microbial profile was different to those observed in preterm fed MOM. Remarkably, preterm infants fed DHM showed closer microbial profiles to preterm fed their MOM. Inferred metagenomic analyses showed higher presence of *Bifidobacterium* genus in mother’s milk group was related to enrichment in the Glycan biosynthesis and metabolism pathway that was not identified in the DHM or in the formula fed groups. In conclusion, DHM favors an intestinal microbiome more similar to MOM than formula despite the differences between MOM and DHM. This may have potential beneficial long-term effects on intestinal functionality, immune system, and metabolic activities.

## Introduction

In preterm infants, neonatal microbial dynamics and alterations in early gut microbiota may precede and/or predispose to diseases such as NEC or LOS (The European Perinatal Health Report, 2010). In the newborn period differential microbial colonization clearly relates to weeks of gestation and mode of delivery (Lehtonen et al., 2017), but also to infant nutrition (Collado et al., 2015).

Human milk is the gold standard for infant nutrition in the first 12 months of life for term and preterm newborn infants (American Academy of Pediatrics, 2012). Beyond nutritional components, HM contains important bioactive compounds such oligosaccharides, cytokines, immunoglobulins, microbes, and proteins among others that directly influence the developing infant and shape the intestinal microbiota colonization. Those bioactive compounds are considered not only protective but also stimulate the development and maturation of the immature immune system (Agostoni, 2010; Ballard and Morrow, 2013). Moreover, breastfeeding practices have been associated with a risk reduction of NEC and LOS in preterm infants (Meinzen-Derr et al., 2009; Collado et al., 2012) and an improvement in growth and cognitive development and modulating metabolic and inflammatory conditions in later childhood and adulthood (Ballard and Morrow, 2013; Belfort et al., 2016). However, often mothers who deliver preterm are not able to successfully breastfeed (Wilson et al., 2017). In the absence of MOM, DHM has become the preferred alternative for preterm infants (ESPGHAN Committee on Nutrition et al., 2013). Despite the beneficial effects of DHM, little is known about its effect upon preterm gut microbiota colonization and its potential biological implications. Most HM bank guidelines recommended Holder pasteurization (62.5°C for 30 min) in order to inactivate viral and bacterial agents (Human Milk Banking Association of North America, 2000; Arslanoglu et al., 2010; National Institute for Health and Clinical Excellence, 2010; Peila et al., 2016). However, HM pasteurization causes the loss of several of the structural and functional properties of HM (Baro et al., 2001). Moreover, pasteurization also significantly reduces the cellular and bacterial constituents, enzymatic activities, and IgA, lactoferrin and lysozyme contents (Untalan et al., 2009; Christen et al., 2013; Espinosa-Martos et al., 2013; Sousa et al., 2014). Contrarily, other components with biological relevance such as oligosaccharides, nucleotides, and polyunsaturated and long chain fatty acids (LCPUFA) are preserved (Bertino et al., 2008; Coscia et al., 2015). As a consequence, pasteurization is still a matter of debate (Bertino et al., 2009; ESPGHAN Committee on Nutrition et al., 2013; Corpeleijn et al., 2016; Madore et al., 2017).

In this scenario, we hypothesized that DHM would promote a specific microbiota profile similar to the observed in the preterm infants who receive MOM. To pursue this objective we analyzed the impact of different nutritional approaches upon the gut microbiota composition of preterm infants born at ≤32 weeks of gestation.

## Materials and Methods

### Study Design

We conducted a prospective, observational unicentric cohort study including consecutively admitted preterm infants born at ≤32 weeks of gestation and birth weight ≤1,500 g in the Division of Neonatology of the University and Polytechnic Hospital La Fe (Valencia, Spain) during a 12-month period. The study protocol was approved by the hospital IRB (Comité de Ética e Investigación Médica) and parents approved and signed informed consent in all cases.

### Patients’ Characteristics

Inclusion and exclusion criteria are shown in **Table 1**. Demographic, perinatal, clinical, and analytical data were recorded and matched according to the type of feeding (**Table 2**). Administration and duration of antibiotic therapy was also collected.

**Table 1:** Inclusion and exclusion criteria for preterm infants receiving different types of nutrition and whose microbiota was studied.

Inclusion criteria	Exclusion criteria
BW ≤ 1.500 g and/or GA ≤ 32 weeks	GA > 32 weeks
Enteral intake (≥ 150 mL/kg/day)	Parents refuse to participate/sign informed consent
The principal nutrient received (MOM, DHM, or formula) represents 80% of the intake	Mixed breastfeeding
DHM from just one donor to one premature or maximum of two different donors	Chromosomopathies
No additional treatments that could alter the microbiota (e.g., probiotics) or oxidative status (e.g., vitamins C, A, and E)	Major malformations or surgery of the digestive tract

*GA, gestational age; BW, birth weight; MOM, mother’s own milk; DHM, donor human milk.*

**Table 2:** Perinatal characteristics and confounders of preterm infants receiving different types of nutrition and whose microbiota was studied.

	MOM (*n* = 34)	DHM (*n* = 28)	*p*-Value
GA weeks, mean (SD)	28.85 (1.9)	29.78 (2.42)	0.09
Antenatal steroids full course (%)	97.1	92.8	0.44
Type of delivery (%)			
Vaginal	58.8	39.3	0.126
Cesarean section	41.2	60.7	
Birth weight (g), mean (SD)	1,228 (301)	1304.3 (262)	0.3
Race (%)			
Caucasian	85.3	67.8	0.1
Non-Caucasian	14.7	32.1	
Apgar 1 min (median; 5–95% CI)	7.3 (2.15)	7.1 (1.81)	0.68
Apgar 5 min (median; 5–95% CI)	9.02 (1.3)	8.6 (1.4)	0.24
Age (days) at sample collection, mean (SD)	9.7 (7.03)	8.7 (6.2)	0.52
Chorioamnionitis (%)	76.4	89.2	0.19
Mechanical ventilation	11.7	14.3	0.76
Non-invasive ventilation	75	85.3	0.3
Persistent ductus arteriosus	29.4	25	0.69
Antibiotic therapy (%)	38.2	39.2	0.93

Fresh DHM was collected and immediately frozen at -20°C until Holder pasteurization process (62.5°C for 30 min followed by fast cooling). After treatment, pasteurized DHM was frozen until the distribution to patients. The Division of Neonatology protocol involves strong support to breastfeeding and offering DHM as a supplement to preterm infants born below ≤32 weeks or ≤1,500 g birth weight. Type of feeding was a parents’ decision. The infants were fed at least with an 80% of either MOM or DHM and table intakes of 150 cc/kg/day.

The nutritional intake was prospectively monitored but never influenced by this observational study.

### Fecal Samples, DNA Extraction, and 16S rDNA Sequencing

Fecal samples were directly collected from the diaper when full enteral feeding (defined as ≥150 cc/kg/day of MOM, DHM, or formula) was achieved. Samples were frozen and stored at -80°C for later analysis.

Total fecal DNA was isolated using the MasterPure Complete DNA & RNA Purification Kit (Epicentre, Madison, WI, United States) according to the manufacturer’s instructions with modifications that included a bead-beater step and enzyme incubation to increase DNA extraction as described elsewhere (Boix-Amoros et al., 2016). Total DNA concentration was measured using a Qubit^®^ 2.0 Fluorometer (Life Technologies, Carlsbad, CA, United States) and normalized to 5 ng/μL for 16S rDNA gene (V3–V4 region) amplification using Nextera XT Index Kit. Amplicons were checked with a Bioanalyzer DNA 1000 chip and libraries were sequenced using a 2 × 300 bp paired-end run (MiSeq Reagent kit v3) on a MiSeq-Illumina platform (FISABIO sequencing service, Valencia, Spain). Controls during DNA extraction and PCR amplification were also included and sequenced.

### Bioinformatics and Statistical Analysis

Data were obtained using an *ad hoc* pipeline written in RStatistics environment (R Core Team, 2012) and data processing was performed using a QIIME pipeline (version 1.9.0) (Caporaso et al., 2010). Chimeric sequences and sequences that could not be aligned were also removed from the data set. The clustered sequences were utilized to construct operational taxonomic units (OTUs) tables with 97% identity and representative sequences were taxonomically classified based on the Greengenes 16S rRNA gene database (version 13.8). Sequences that could not be classified to domain level, or classified as cyanobacteria and chloroplasts, were removed from the data set. Subsequently, alpha diversity indices (Chao1 and Shannon, Species richness estimates and diversity index, respectively), beta diversity based on UNIFRAC unweighted distance (phylogenetic) and Bray–Curtis distance (non-phylogenetic), and PERMANOVA with 999 permutations was used to test significance between groups. The DESeq2 method was used to determine differential abundances of specific bacteria between feeding groups. Calypso software^1^ version 7.36 was used with total sum normalization for the statistical analysis, and also, cumulative sum scaling (CSS) normalization for multivariate test. Furthermore, predictive inferred functional analysis was performed using PICRUSt (Phylogenetic Investigation of Communities by Reconstruction of Unobserved States) approach as described (Langille et al., 2013). Linear discriminant analysis effect size (LEfSe) (Segata et al., 2011) was used to detect unique biomarkers in relative abundance of bacterial taxonomy and specific functions (KEGG pathways). A size effect cut-off of 3.0 on the logarithmic linear discriminant analysis (LDA) score was used. The 16S rRNA gene sequence data generated is available through GenBank Sequence Read Archive Database under project accession number PRJEB25948.

## Results

### Patients’ Characteristics

A total of 69 preterm infants ≤32 weeks of gestation pertaining to the MOM (*n* = 34), DHM (*n* = 28), and formula (*n* = 7) groups were recruited. No differences for prenatal demographic characteristics or confounders during the hospitalization between patients in the three feeding-type groups were found (**Table 2**). Individual information is available in Supplementary Table S1.

A small group of preterm neonates (*n* = 7) fed with formula milk was also included. The low number is explained because the protocol of our NICU recommends for all preterm ≤32 weeks of gestation and ≤1,500 g MOM and DHM as an alternative and therefore preterm fed with formula render exceptional. Characteristics of the formula group were as follows: 33 ± 2 weeks of gestation; 1,702 ± 321.6 g birth weight; 57.1% male; 14.3% born by vaginal delivery (85.7% by cesarean section); 57.1% received antenatal steroids.

### Impact of DHM on Preterm Microbiota

**FIGURE 1:**
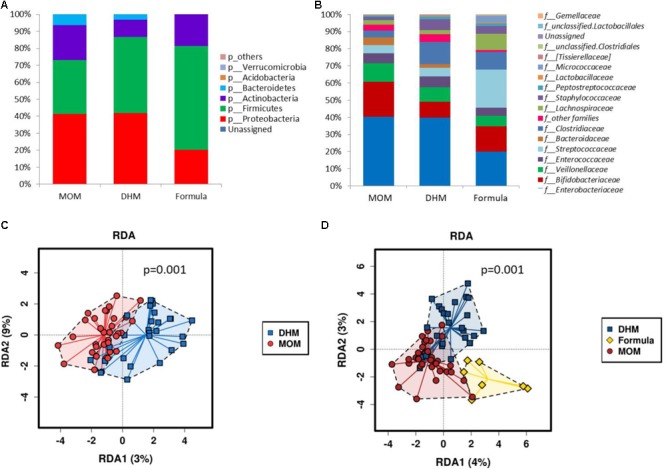
Microbial relative abundances (%) at phylum **(A)** and family level **(B)** of preterm gut microbiome according to diet (MOM, DHM, and formula). RDA plots of the preterm microbiota grouped by infant feeding type: MOM vs DHM **(C)** and MOM, DHM, and formula **(D)**.

Significant differences were found in preterm microbiota composition according to feeding type. We found lower relative abundance of Firmicutes (30.9 vs 45.5%, *p*-value = 0.029) and higher abundance of Actinobacteria (20.1 vs 10.2%, *p*-value = 0.040) in MOM compared to DHM group (**Figure 1A**). At family level, higher abundance of Bifidobacteriaceae (19.5 vs 9.0%, *p*-value = 0.027) and lower abundance of Clostridiaceae (3.7 vs 11.2%, *p*-value = 0.029) were observed in MOM compared to DHM (**Figure 1B**). At genus level, higher levels of *Bifidobacterium* (19.5 vs 8.98%, *p*-value = 0.027) and unclassified Enterobacteriaceae (29.77 vs 18.48%, *p*-value = 0.060) and lower levels of *Citrobacter* (2.60 vs 9.83%, *p*-value = 0.060), and unclassified Clostridiaceae (3.46 vs 9.43%, *p*-value = 0.062) were observed in fecal samples of MOM as compared to the DHM group.

The effect of the diet on the preterm gut microbiota was explored by applying the multivariate method PERMANOVA with 999 permutations on the phylogenetic distances (*p*-value = 0.09 for unweighted UNIFRAC distance) and Bray–Curtis distance (non-phylogenetic; *p*-value = 0.0046). Furthermore, multivariate redundant discriminant analysis (RDA) based on the observed OTUs showed statistically significant differences in microbial composition between MOM and DHM groups (*p* = 0.001) (**Figure 1C**).

**FIGURE 2:**
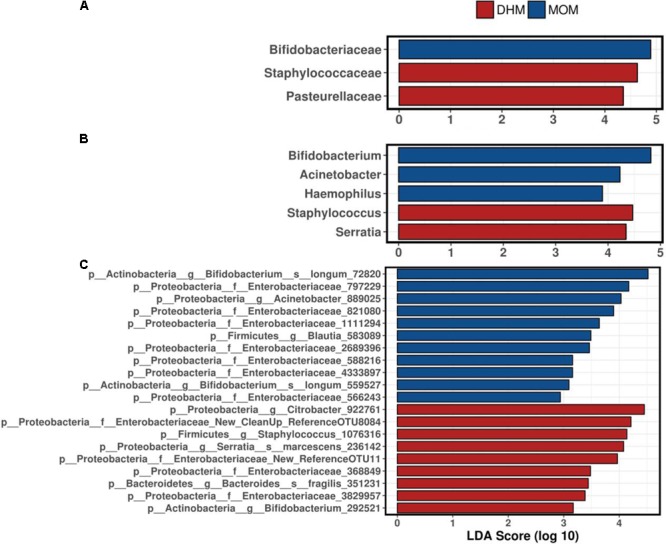
Linear discriminant analysis (LDA) combined with effect size measurements (LEfSe) revealed specific microbes at family **(A)**, genus **(B)**, and OTUs **(C)** between MOM and DHM feeding groups.

To explore the variation of the microbial community composition between MOM and DHM, we performed LEfSe tests to detect differences in relative abundance of bacterial taxa across fecal samples (**Figure 2**). At the family level, Bifidobacteriaceae family was significantly enriched in MOM compare to DHM samples (LDA = 4.90, *p*-value = 0.025) while Staphylococcaceae (LDA = 4.63, *p*-value ≤0.042). Pasteurellaceae family was enriched in DHM (LDA = 4.358, *p*-value = 0.050). Specific enriched features at genus and OTUs levels are shown in **Figure 2**.

DESeq2 test was used to identify differential abundances of specific bacteria between feeding groups. Actinobacteria phylum was higher in MOM compared to DHM group (20.07 vs 10.25%, *p*-value = 0.0044, FDR = 0.013). The abundance of *Staphylococcus* (*p*-value <0.0001, FDR < 0.0001), *Clostridium* (*p*-value <0.0001, FDR = 0.0013), *Serratia* (*p*-value <0.0001, FDR = 0.0022), *Coprococcus* (*p*-value = 0.0021, FDR = 0.012), *Aggregatibacter* (*p*-value = 0.015, FDR = 0.059), and *Lactobacillus* (*p*-value = 0.056, FDR = 0.18) was significantly higher in DHM group than MOM group. However, *Bacteroides* (*p*-value <0.0001, FDR = 0.0044), *Acinetobacter* (*p*-value <0.0001, FDR = 0.002), and *Haemophilus* (*p*-value = 0.0014, FDR = 0.009) were significantly higher in the MOM than in DHM group.

### Impact of Formula vs Human Milk Groups on Preterm Microbiome

Despite the low number of formula fed preterm infants, we analyzed the differences in microbiome between MOM, DHM, and FM groups. A multivariate RDA based on the observed OTUs showed statistically significant differences in microbial composition between groups (*p* = 0.001) (**Figure 1D**). Significantly higher relative abundance of Firmicutes (*p*-value = 0.027, FDR = 0.016) was observed in FM group compared to MOM and DHM (**Figure 1A**). At genus level, significant higher abundance of *Blautia* (*p*-value <0.001, adjusted *p*-value = 0.033, FDR = 0.033), *Streptococcus* (*p*-value = 0.0024, FDR = 0.054), *Acidaminococcus* (*p*-value = 0.0093, FDR = 0.099), *Rothia* (*p*-value = 0.0059, FDR = 0.088), and *Dorea* (*p*-value = 0.011, FDR = 0.099) were observed in the FM group compared to the MOM and DHM groups.

**FIGURE 3:**
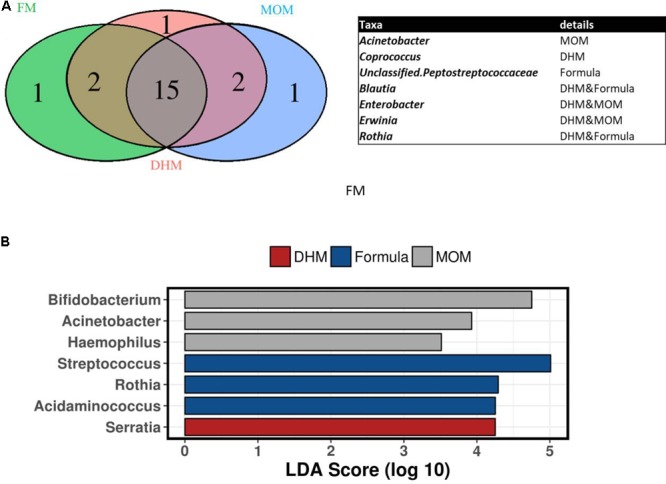
Venn diagram of shared genus between group **(A)** and linear discriminant analysis LEfSe test at genus level between MOM, DHM, and formula preterm microbiota **(B)**.

Preterm core microbiome was composed by a total of 15 shared genus independently of feeding-type diet (**Figure 3A**). *Acinetobacter* genus was exclusively present in MOM group; while *Coprococcus* genus was present in DHM and unclassified Peptostreptococcaceae genus in formula group.

Linear discriminant analysis effect size test showed that *Rothia, Streptococcus*, and *Acidaminococcus* genus were significantly enriched in formula group compared to MOM and DHM group, while *Bifidobacterium, Acinetobacter*, and *Haemophilus* genus were enriched significantly in MOM compared to DHM, representing a hallmark for breast fed preterm gut microbiota (**Figure 3B**).

**FIGURE 4:**
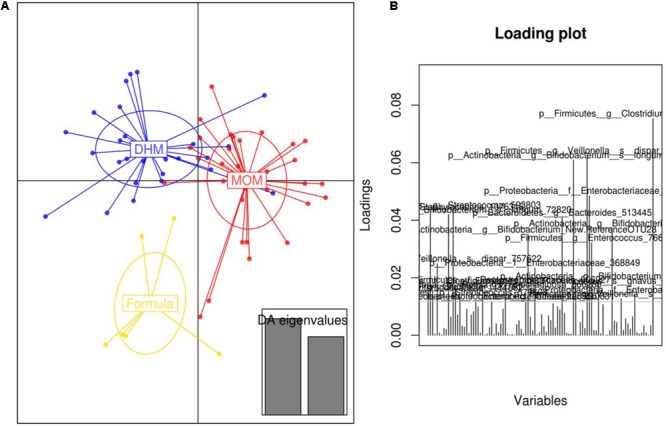
Discriminant analysis of principal components (DAPC) plot **(A)** at OTUs level revealed distinct clustering of the MOM (*red*), DHM (*blue*), and formula-fed group (*yellow*). Canonical loading plot **(B)** showing differentially abundant bacterial genera. The individual peaks show the magnitude of the influence of each variable on separation of the groups (0.05 threshold level).

We also applied discriminant analysis of principal components (DAPC) identifying specific feeding-type related clusters of preterm microbiota (**Figure 4**). These microbial shifts were attributed to subtle changes in the abundance of several bacterial OTUs. *Clostridium, Bifidobacterium*, unclassified Enterobacteriaceae and *Veillonella* related OTUs were the strongest indicator of the presence of distinct microbial clusters.

### Functional Assignment of the Preterm Microbiota

**FIGURE 5:**
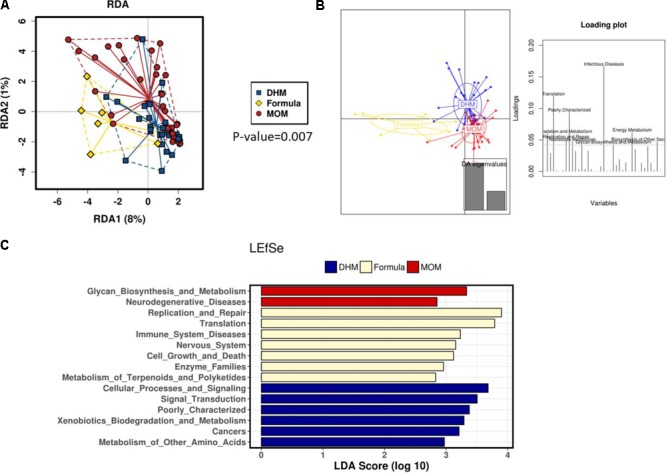
RDA plot **(A)**. Discriminant analysis of principal components (DAPC) plot and loading plot **(B)** at KEGG pathways. The individual peaks in the loading plot show the magnitude of the influence of each variable on separation of the groups (0.05 threshold level). LEfSe test **(C)** revealed distinct clustering of the MOM (*red*), DHM (*blue*), and formula-fed group (*yellow*).

Inferred metagenomic PICRUSt prediction revealed significant differences in the main functional classes (Kyoto Encyclopedia of Genes and Genomes, KEGG categories at level 2), deriving from functional acquisitions associated with different diets (multivariate RDA test, *p* = 0.007) (**Figure 5A**). Moreover, no different metabolic profile was found (RDA test *p*-value >0.05) when MOM and DHM were compared, while formula functional profile was significantly different from those observed in MOM (RDA test *p*-value = 0.024) and DHM (RDA test *p*-value = 0.002).

The DAPC to identify specific clusters of functional activity (KEGG level 2 and 3) of the gut microbiome in preterm groups according to type of diet (**Figure 5B**) suggesting a distinct KEGG activities DHM and MOM microbiome activities are similar than those observed in the FM group.

Linear discriminant analysis effect size analysis performed on PICRUSt output showed several KEGG (level 2, **Figure 5C** and level 3, Supplementary Figure S1) categories differentially present in the MOM, DHM, and formula groups. MOM functional profile is mostly represented by bacterial secretion system, lipopolysaccharide (LPS) biosynthesis, and biosynthesis. In particular, we observed a deprivation in functions involved in complex carbohydrate metabolism, deriving from HMO present in breast milk, such us Glycan biosynthesis and metabolism in the formula group (*p*-value = 0.019) compared to the other MOM and DHM groups. This glycan pathway was not different between MOM and DHM profiles (*p*-value >0.05). Interestingly, we observed a reduced LPS biosynthesis (*p*-value <0.0001) and LPS biosynthesis proteins (*p*-value <0.0001) in formula fed infants compared to MOM and DHM (Supplementary Figure S2). However, the most predominant function in DHM group was the two-component regulatory system followed by other functions related to amino acid metabolisms, fatty acids metabolisms (butanoate metabolism) and to sulfur metabolism and sulfur relay system and also, nitrogen metabolism in DHM group (Supplementary Figure S2). Methane metabolism pathway is also enriched in formula group as compared to MOM and DHM groups (Supplementary Figure S2). Furthermore, we also observed in formula group an enrichment of KEGG functions related to sugar metabolisms as galactose metabolism, and amino sugar and nucleotide sugar metabolisms compared to the observed ones in MOM and DHM (Supplementary Figure S2).

## Discussion

In recent years, nutritional practices have shifted toward encouraging breastfeeding practices in preterm neonates (Keunen et al., 2015; O’Connor et al., 2016). In the absence of MOM, DHM has become the preferred nutritional alternative and a formula feeding remains the last option when the others are not available. The setting up of milk banks has rendered DHM the most widely prescribed alternative to MOM (Underwood, 2013).

In preterm infants, our results have demonstrated that the feeding type has an important impact on gut microbial composition in preterm infants ≤1,500 g. We found that MOM and DHM microbial profiles were different. MOM fed babies showed a significantly enriched and greater presence of Bifidobacteriaceae and lower of Staphylococcaceae, Clostridiaceae, and Pasteurellaceae compared to DHM fed babies. At genus level, higher levels of *Bifidobacterium* and unclassified Enterobacteriaceae and lower unclassified Clostridiaceae were observed in fecal samples from MOM group compared to DHM preterm group. It has been reported that preterm infant receiving MOM had a higher abundance of Clostridiales, Lactobacillales, and Bacillales compared to both the DHM and formula groups (Gregory et al., 2016). Both these groups had higher abundance of Enterobacteriales. After controlling for gender, postnatal age, weight, and birth gestational age, the diversity of gut microbiota increased over time and was constantly higher in infants fed MOM relative to infants with other feeding types. Finally, in the formula microbial profile was distinct than those observed in MOM and DHM, suggesting that DHM favors an intestinal microbiome more similar to MOM despite the differences between MOM and DHM.

In accordance to the microbiota shifts, we observed that KEGG profiles in DHM and MOM were similar than those microbial profile observed in formula. MOM functional profile is mostly represented by bacterial secretion system, LPS biosynthesis and biosynthesis protein which would be mainly related to the presence of Gram-negative bacteria. Interestingly, we observed a significant reduction on the LPS biosynthesis and proteins in formula fed infants compared to MOM and DHM (without difference between them). These data would suggest the potential link between LPS and immune response as reported previously (Cullen et al., 2015). Recent study has been shown that variation on the microbial LPS produced by specific microbiota groups as Enterobacteriaceae and *Bacteroides* spp., could either stimulate or actively inhibit inflammatory pathway and also, have a role on the autoimmune diseases risk (Vatanen et al., 2016). In our context, MOM and DHM modulate a preterm gut microbiota toward an enrichment in *Bifidobacterium* spp. and also, *Bacteroides* spp. that may promote the specific LPS signaling and its contribution to the immune system. Furthermore, we observed a significant reduction in functions derived from the presence of HMO and involved in complex carbohydrate metabolism (e.g., glycan biosynthesis) in the FM group as compared with the MOM and DHM groups. These differences could be explained by the abundance of HMO metabolizers as *Bifidobacterium* and *Bacteroides* spp. in preterm gut fed with MOM and DHM compared to formula fed preterm.

We also found enrichment on the functions related to the fatty acids metabolism and to sulfur and nitrogen metabolism in DHM and MOM groups. Several enteric bacteria and oral bacteria produce reduced sulfur and nitrogen and maybe some specific bacteria, as Deltaproteobacteria, *Clostridium* spp., *Veillonella* spp., *Rothia* spp., would be responsible for this functional contribution as they were enriched in DHM group although it was also observed in MOM group. In formula metagenome, we observed enrichment of KEGG functions related to sugar metabolisms as galactose metabolism, involved in conversion of galactose into glucose could arise from consumption of infant formula and/or dairy products, and amino sugar and nucleotide sugar metabolisms. In general, minor differences were observed in the functional profiles between MOM and DHM suggesting the potential effect of DHM in mimicking the microbiome functionality of own maternal milk feeding. These results would open new possibilities in future research where bigger studies should be carried out.

Two important factors influence the differences found in preterm gut microbiota depending on feeding type (MOM and DHM). The first one would be related to the timing of milk extraction in relation to gestational age and to the lactation stage. While MOM is the biological product of a prematurely interrupted gestation, DHM is composed mainly by donated mature milk from mothers who completed term gestations and were extracting milk for several weeks thereafter. Preterm milk has higher amount of proteins, fats, and energy (Underwood, 2013; Gidrewicz and Fenton, 2014). Hence, depending on the staging of lactation they can be also a great variability among donators of components such as essential fatty acids or amino acids indispensable not only for an adequate nutrition but also for promoting microbiota colonization. Although donor milk pooling tries to avoid these circumstances, a recent study has shown a shortage of docosahexaenoic acid or lysine in DHM. Targeted supplementation would be needed not only to optimize nutritional properties of DHM but also to improve bacterial colonization (Ballard and Morrow, 2013). The second factor would be related with the pasteurization procedure that inevitably alters essential thermolabile milk components. Hence, differences in the microbiota would be at least partially explained by the different composition in relation to nutritional parameters and bioactive compounds such as immune markers, microbiota, oligosaccharides, neurotrophic, and growth factors among others (Bertino et al., 2008; Untalan et al., 2009; Ballard and Morrow, 2013; Christen et al., 2013; Espinosa-Martos et al., 2013; Sousa et al., 2014; Coscia et al., 2015; Valentine et al., 2017). Altogether these findings could explain the similarities and differences in the microbiota profile between preterm infants fed DHM or MOM and influence health outcomes in preterm infants.

Finally, the number of formula fed infants included in this study was limited (*n* = 7) due mainly because milk bank provides with DHM to almost all preterm babies attended in our NICU (>90%) and therefore it is difficult to recruit preterm babies on formula feeding and randomization was not ethically acceptable. Despite these limitations, our results reveal a substantial impact of DHM feeding on the structure of the intestinal microbial community composition.

## Conclusion

Feeding type modulates the preterm microbiome composition. DHM feeding had an impact on preterm microbiota that could have potential beneficial long-term effects on intestinal functionality, immune system, and metabolism. However, available pasteurization methods cause changes that may blunt many of the positives aspects derived from the use of MOM. Therefore, further studies are stringently need to understand the complex links between microbiome and DHM host, its impact on health programming, and to develop sensitive methods capable of providing promptly after birth preterm infants with HM as similar as possible to their MOM when the latter is not yet available.

## Author Contributions

AP-L, MG, MC, MCC, and MV planned the experiments. AP-L, MG, MC, AN-R collected the samples and clinical data. AP-L, CA, and MCC performed the microbiological analysis and analyzed data. AP-L wrote first draft and all authors commented criticized, and reviewed the manuscript. All authors accepted the final version the manuscript.

## Conflict of Interest Statement

The authors declare that the research was conducted in the absence of any commercial or financial relationships that could be construed as a potential conflict of interest.

## Footnotes

1 http://cgenome.net/calypso/

## Abbreviations

BPD: bronchopulmonary dysplasia
DHM: donor human milk
FM: special preterm formula
HM: human milk
LOS: late onset sepsis
MOM: mother’s own milk
NEC: necrotizing enterocolitis
NICU: neonatal intensive care unit
PVL: periventricular leukomalacia
ROP: retinopathy of prematurity
