# Supplementary figures and images for: Preterm Gut Microbiome Depending on Feeding Type: Significance of Donor Human Milk
Source: Front Microbiol. 2018 Jun 27;9:1376. doi: 10.3389/fmicb.2018.01376 (PMC6030370; doi:10.3389/fmicb.2018.01376)

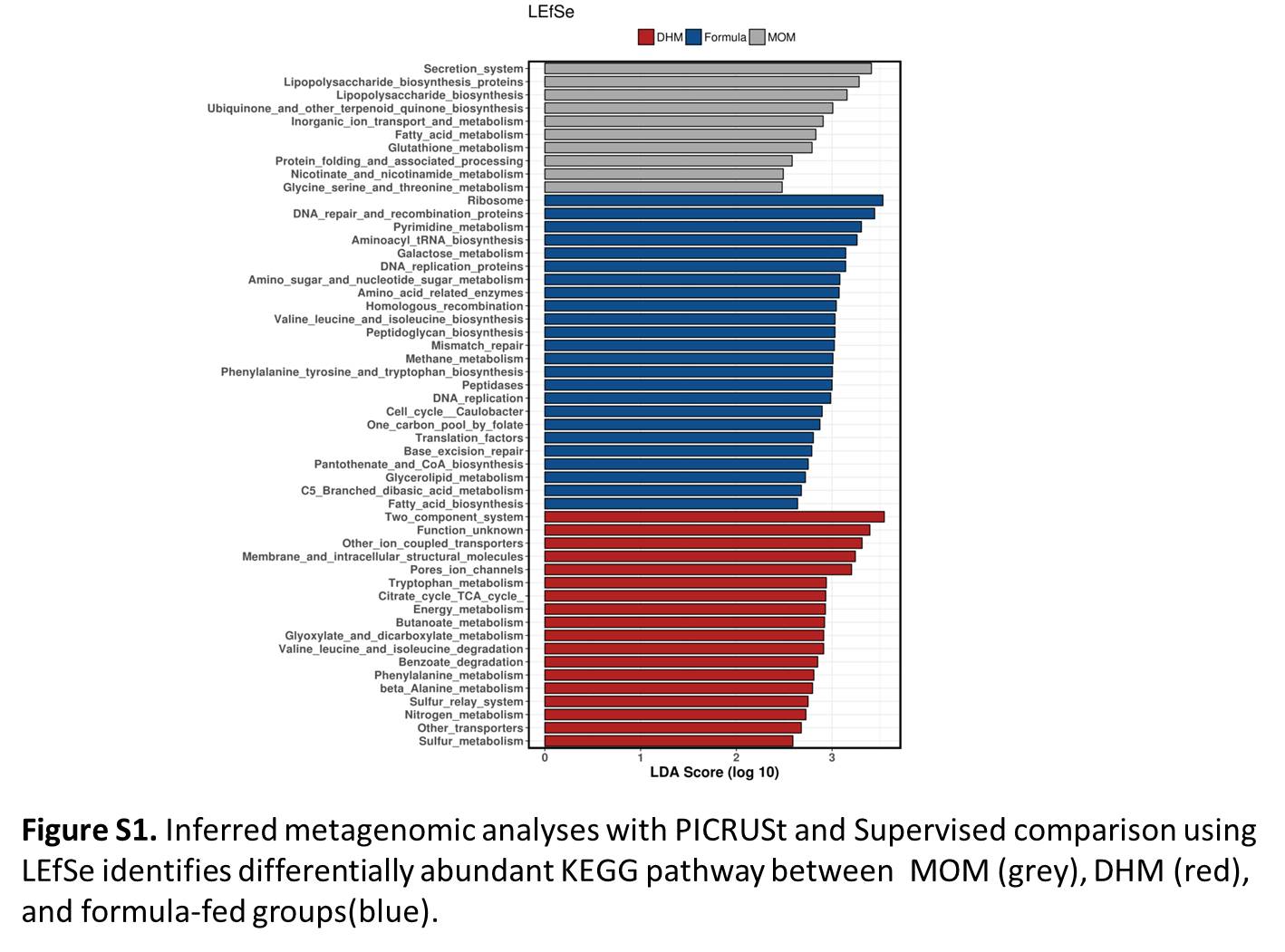

Supplement: Supplementary file 2 [file Image_1.JPEG]

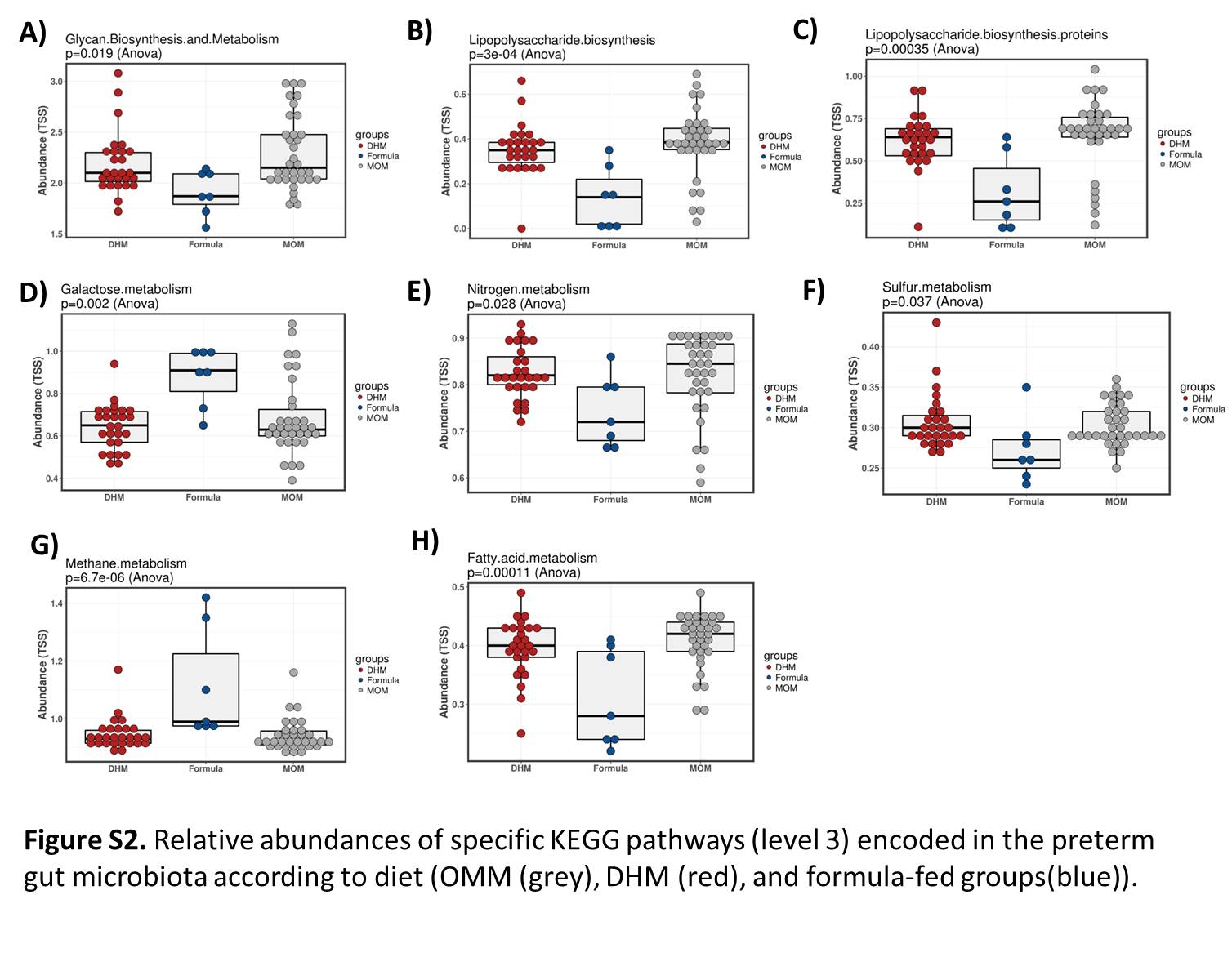

Supplement: Supplementary file 3 [file Image_2.JPEG]
